# Parathyroid Hormone, Cognitive Function and Dementia: A Systematic Review

**DOI:** 10.1371/journal.pone.0127574

**Published:** 2015-05-26

**Authors:** Ilianna Lourida, Jo Thompson-Coon, Chris M. Dickens, Maya Soni, Elżbieta Kuźma, Katarina Kos, David J. Llewellyn

**Affiliations:** 1 The National Institute for Health Research (NIHR) Collaboration for Leadership in Applied Health Research and Care South West Peninsula (PenCLAHRC), University of Exeter Medical School, University of Exeter, Exeter, United Kingdom; 2 University of Exeter Medical School, University of Exeter, Exeter, United Kingdom; University of Glasgow, UNITED KINGDOM

## Abstract

**Background:**

Metabolic factors are increasingly recognized to play an important role in the pathogenesis of Alzheimer’s disease and dementia. Abnormal parathyroid hormone (PTH) levels play a role in neuronal calcium dysregulation, hypoperfusion and disrupted neuronal signaling. Some studies support a significant link between PTH levels and dementia whereas others do not.

**Methods:**

We conducted a systematic review through January 2014 to evaluate the association between PTH and parathyroid conditions, cognitive function and dementia. Eleven electronic databases and citation indexes were searched including Medline, Embase and the Cochrane Library. Hand searches of selected journals, reference lists of primary studies and reviews were also conducted along with websites of key organizations. Two reviewers independently screened titles and abstracts of identified studies. Data extraction and study quality were performed by one and checked by a second reviewer using predefined criteria. A narrative synthesis was performed due to the heterogeneity of included studies.

**Results:**

The twenty-seven studies identified were of low and moderate quality, and challenging to synthesize due to inadequate reporting. Findings from six observational studies were mixed but suggest a link between higher serum PTH levels and increased odds of poor cognition or dementia. Two case-control studies of hypoparathyroidism provide limited evidence for a link with poorer cognitive function. Thirteen pre-post surgery studies for primary hyperparathyroidism show mixed evidence for improvements in memory though limited agreement in other cognitive domains. There was some degree of cognitive impairment and improvement postoperatively in observational studies of secondary hyperparathyroidism but no evident pattern of associations with specific cognitive domains.

**Conclusions:**

Mixed evidence offers weak support for a link between PTH, cognition and dementia due to the paucity of high quality research in this area.

## Introduction

In the absence of disease modifying or curative treatments, the identification of potentially modifiable risk factors for Alzheimer’s disease and other dementia subtypes is particularly important. Parathyroid hormone (PTH) is of interest in relation to cognitive function and dementia as it crosses the blood brain barrier and PTH receptors are found throughout the human brain [1]. PTH regulates circulating and intracellular calcium levels, and may induce apoptosis due to calcium overloading [2]. Elevated PTH levels are associated with reduced regional cerebral blood flow [3], whereas PTH-related protein (PTHrP) inhibited calcium channel activity via the PTH/PTHrP receptor may contribute to maintaining normal neuronal function (for example by increasing resistance to excitotoxic injury) [4]. PTH also promotes the conversion of vitamin D to its active form (1,25-dihydroxyvitamin D) [5], which an emerging body of evidence suggests may be neuroprotective [6, 7].

Hyperparathyroidism, characterized by elevated/high PTH levels, has been associated with many chronic conditions including impaired cognitive function and dementia [8–11]. Patients with primary hyperparathyroidism (high PTH and calcium levels), the most common parathyroid disease, often report cognitive complaints and observational studies have described poorer cognitive function in those patients compared to control groups including impaired performance in memory and attention tasks [12, 13]. Although not supported by all studies [14, 15], there is evidence from surgical studies that parathyroidectomy, which if successful leads to normalization of PTH and calcium levels, is associated with significant improvement in cognitive function [9, 16, 17] or even reversibility of cognitive deficits [12]. Secondary hyperparathyroidism is determined by high PTH levels, normal to low calcium levels and concurrent decrease in vitamin D levels. Failing kidneys may cause secondary hyperparathyroidism and therefore the condition is frequently occurring in chronic kidney disease. Cognitive impairment has also been observed in this parathyroid condition [18] followed by postoperative improvement [10]. Moreover, recent findings suggest that elevated PTH levels may be associated with increased risk of cognitive decline and incident dementia [19]. Elevated PTH concentrations in dementia patients have been documented as well [20, 21]. There is some indication that hypoparathyroidism (low serum PTH and calcium levels) may be associated with poorer cognitive function too [22, 23]. Existing narrative reviews [24, 25] are inconsistent and do not provide a comprehensive picture as they have focused on primary hyperparathyroidism and the effects of surgery on cognition. Even though the need to understand the nature of the associations between hyperparathyroidism and cognition or indeed PTH as a potential index of cognitive impairment or dementia has been recognized [26], there is no systematic evaluation of all the available evidence in this area to inform current knowledge and future research needs. Therefore, our aim was to conduct a systematic review to update and evaluate the current research for the association of PTH and parathyroid conditions with cognitive function and dementia.

## Methods

### Literature search

The systematic review was conducted following the general principles published by the NHS Centre for Reviews and Dissemination [27]. A predefined protocol was developed following consultation with experts in the field (S1 Text). The following electronic databases were searched for relevant studies from inception to January 2014: Medline, Embase, PsycINFO, HMIC, CINAHL, AMED, Web of Science, and the Cochrane Library of Systematic Reviews. The search strategies used text words and relevant indexing (MeSH terms) to identify studies on the association between parathyroid hormone, cognition function and dementia. The search terms parathyroid, hyperparathyroidism, parathormone, cognitive (function or decline or impairment or assessment or change), memory, neuropsychological (assessment or evaluation or test), Alzheimer, and (vascular or frontotemporal or Lewy bodies) dementia were used (full strategy in supplementary data). Forward and backwards citation searching was used to identify any additional relevant studies. The journal *Surgery* (January 1995-January 2014) and the *Journal of Clinical Endocrinology & Metabolism* (January 1997-January 2014) were electronically “hand”- searched, having been identified as important journals in the field. Internet searches were performed on the following websites: Alzheimer’s Society (www.alzheimers.org.uk), Alzheimer’s Research UK (www.alzheimersresearchuk.org), Alzheimer’s Association (www.alz.org) and Hypoparathyroidism UK (www.hpth.org.uk). The section of each website labeled “Research” or research related tabs (e.g. ‘research portfolio’, ‘researchers and professionals’) were reviewed in detail (IL).

### Study selection

Studies were included if they investigated the association of parathyroid hormone with cognitive function or dementia measured by widely used neuropsychological tests or international diagnostic criteria. Studies evaluating the relationship between parathyroid hormone and cognitive variables assessed only by self-report measures were excluded from the systematic review, as were case-reports, narrative reviews, letters and editorials that did not include original research findings. There was no restriction in study design or language of publication. The titles and abstracts retrieved by the electronic search were screened independently by two reviewers (IL & MS). The full text of potentially relevant papers was retrieved and screened the same way. Any discrepancies were resolved with the involvement of a third reviewer (DJL).

### Data extraction and quality assessment

A customized data extraction form with versions to match different study designs was developed, pilot tested on four included studies and refined accordingly. Data on study and participant characteristics, intervention/exposure, parathyroid hormone, covariates, method of cognitive assessment and relevant outcomes were extracted by one reviewer (IL) and checked by the second reviewer (MS). We attempted to contact corresponding authors of studies where relevant data was not reported; one author responded but was not able to provide additional data [35]. The quality of the design and reporting of included studies was assessed in the same way using the Downs and Black checklist, a reliable and valid quality index for the appraisal of both randomized and non-randomized studies [28]. Discrepancies were resolved through discussion (IL & MS).

### Data synthesis

The main findings have been summarized in narrative form. A meta-analysis was not possible due to great variability in cognitive assessment tools and omitted or underreported relevant data. The results are categorized broadly by nature of exposure (i.e. surgical and non-surgical studies) and type of parathyroid condition. Within each condition synthesis is presented by study design and further by the most commonly assessed cognitive outcomes across studies. These included global cognitive function, memory, executive function, attention, dementia and a category for tests assessing other cognitive domains (for example, visuospatial skills, verbal fluency or other). For studies reporting more than one type of research design (i.e. pre-post surgery and baseline case-control comparisons), the more robust study design was included in the synthesis (additional relevant results for each study can be found in S1 Table. A p value < 0.05 was considered statistically significant. One study provided inadequate data for pre-post comparisons and since it was difficult to estimate the nature of changes, a measure of cognitive impairment constructed by the authors expressed in frequencies was used in the synthesis of results.

## Results

**Fig 1:**
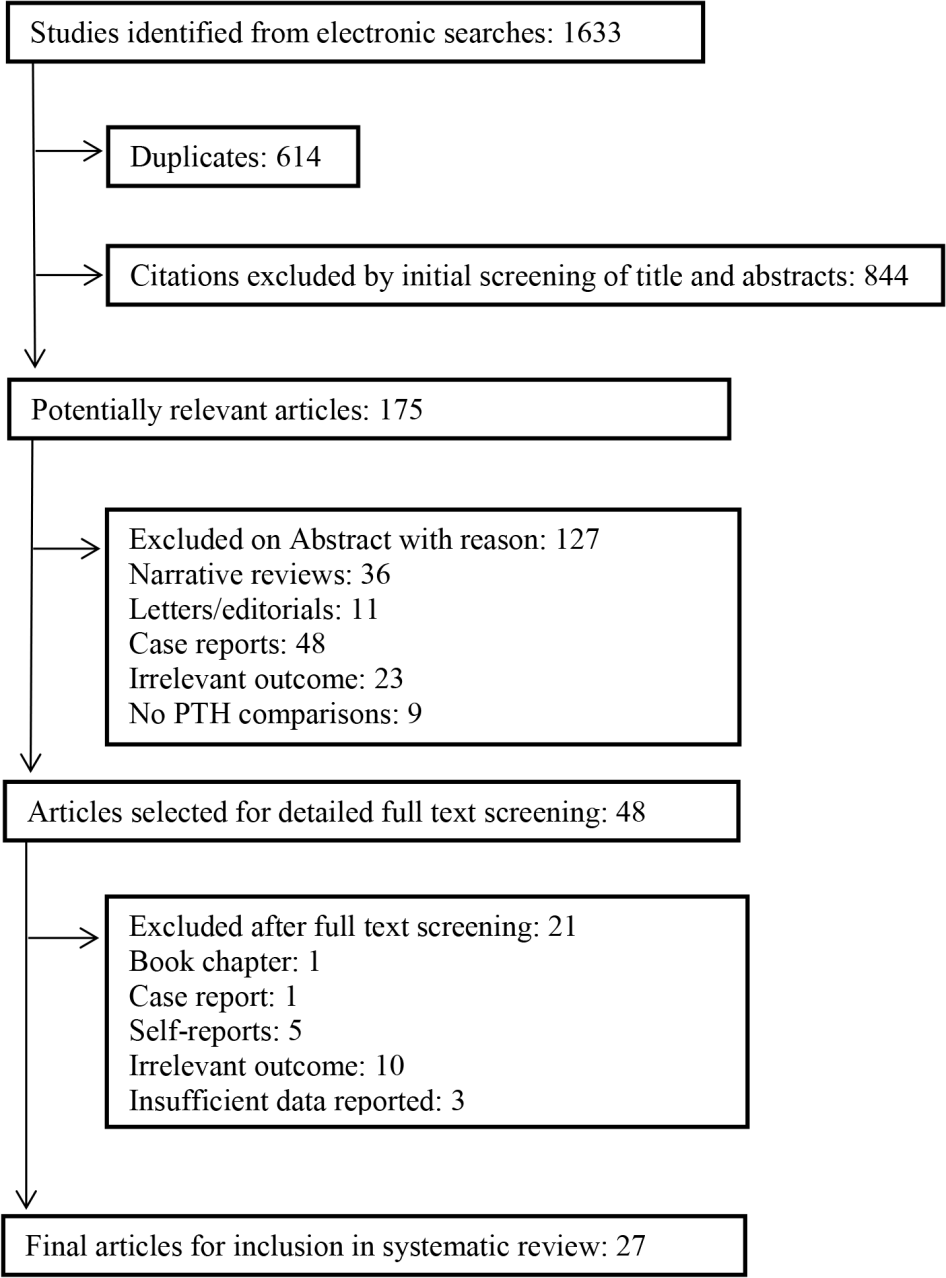
Flow diagram of the selection process of studies included in the systematic review.

The systematic search of all databases yielded a total of 1633 references. Duplicates were checked and excluded (n = 614). Title and abstract screening resulted in the exclusion of a further 971 papers. Full texts of the remaining 48 papers were obtained for detailed review. Twenty-one papers were excluded following full text screening: one case-report, five studies based on self-report measures of cognitive symptoms and three meeting abstracts with insufficient data for analysis; ten further papers were excluded as they did not actually report comparisons between PTH levels or parathyroid conditions and cognitive function or dementia. No additional relevant papers were identified via the supplementary searches (forward and backwards citation, journal ‘hand’-searches and website searches). Twenty-seven papers were eligible for inclusion (Fig 1).

### Study characteristics

Study characteristics are shown in Table 1. Studies were published between 1978 and 2013; most of them were conducted in Europe and the United States with the remainder in Australia and Asia. Study designs identified were: one randomized controlled trial [15], one prospective study [19], fourteen observational studies pre- and post parathyroidectomy [9, 10, 12–14, 16, 17, 29–35], four cross-sectional [18, 36–38] and seven case-control studies [20–23, 39–41]. One study reported data for two parathyroid conditions [29]. Population sizes ranged from 10 to 457 individuals and involved adults mainly recruited from hospitals and endocrine surgery units. The majority of studies included middle-aged populations (50 years or older). Four studies included only women [12, 20, 21, 40] and one study included only men [29]. There were two studies [13, 19] with a follow-up period longer than 6 months, whereas most studies had short-term or medium-term follow-up (less than 6 months).

**Table 1:** Key characteristics of studies relating to PTH levels, parathyroid conditions, cognitive function and dementia.

Exposure and study	Study design; follow-up length	Country	Sample size, N	Mean age (years)	Outcomes and cognitive domains examined	Covariates adjusted for in analysis
**Surgical intervention studies**						
**Primary hyperparathyroidism**						
Perrier [15]	RCT; 6 weeks, 6 months	USA	18	Age range 54–83	Cognitive impairment in memory, attention, processing speed, executive functions	NR
Chiang^a^[14]	Pre-post; 30–380 days	Australia	40	52	Cognitive impairment in memory, attention, processing speed, executive functions	NR
Cogan [29]	Pre-post;4 months	USA	12	Cases: 59 Controls: 54	Cognitive impairment in global cognitive function, memory, attention, visuospatial skills, executive functions	NR
Dotzenrath^a^ [16]	Pre-post; 6 months	Germany	52	Cases: 62 Controls: 61	Cognitive impairment in memory, attention, visuospatial skills, executive functions	NR
Goyal^a^ [29]	Pre-post; 1 & 6 weeks, 3 & 6 months	India	39	Age range 18–60	Cognitive impairment in global cognitive function & memory,	NR
Numann^a^ [17]	Pre-post; 4 months	USA	20	Cases: 63 Controls: 56	Cognitive impairment in memory, attention, verbal abilities, visuospatial skills, planning	NR
Roman^a^ [9]	Pre-post & Case control; 2–4 weeks	USA	41	Cases: 59 Controls: 51	Cognitive impairment in memory & learning	Age, gender, serum calcium^e^, TSH levels
Walker^a^ [12]	Pre-post; 6 months	USA	128	Cases: 61 Controls: 56	Cognitive impairment in memory, attention, intellectual ability, nonverbal abstraction	Age, sex, education, estimated IQ, anxiety, depression
Babinska^a^ [13]	Pre-post; 12–18 months	Poland	70	Cases:52 Controls: 52	Cognitive impairment in memory, attention, executive functions, flexibility and planning, verbal fluency	Depression
Casella [34]	Pre-post^b^; 1 month	Italy	16	72	Global cognitive function/impairment in MMSE	Age, education
Mittendorf^a^ [33]	Pre-post^b^; 4 weeks	USA	47	63	Cognitive impairment in attention, processing speed, executive function	NR
Prager[32]	Pre-post^b^; 6, 12 weeks	Austria	20	61	Cognitive impairment in memory, attention/concentration	Age, gender, serum calcium^e^
Roman [31]	Pre-post^b^; 1, 3 & 6 months	USA	159	60	Cognitive impairment in memory & learning	NR
Benge [35]	Pre-post^b^; 1 month	USA	67	61	Cognitive impairment in memory, learning, attention, processing speed, executive functions, verbal fluency, dexterity	NR
**Secondary hyperparathyroidism**						
Chou^a^ ^,^ ^d^[10]	Pre-post; 12, 16 weeks	Taiwan	62	Cases: 56 Controls: 60	Global cognitive function/impairment in MMSE/CDR	NR
Cogan^d^ [29]	Pre-post; 4 months	USA	11	Cases: 51 Controls: 54	Cognitive impairment in global cognitive function, memory, attention, visuospatial skills, executive functions	NR
**Non-surgical studies**						
**Serum PTH levels**						
Björkman^a^ [19]	Prospective; 1, 5, 10 years	Finland	MMSE/CDR433/457-/304138/141	81	Cognitive decline in MMSE Dementia rating in CDR	Age, gender, baseline cognition, serum ionized calcium, creatinine, ApoE ε4
Kalaitzidis^d^ [36]	Cross-sectional	Greece	256	58^c^	Cognitive impairment in MMSE, Clock-drawing test	Age, CKD stage, diabetes mellitus, PTH
Ogihara^a^ [21]	Case-control	Japan	60	Non-dementia: 80AD: 81, VaD: 80	Alzheimer’s disease, Vascular dementia, cognitive impairment	NR
Johansson [39]	Case-control	Sweden	69	AD: 74Other dementias:74 MCI: 72 Controls: 75	Alzheimer’s disease, Vascular dementia, other dementias, stable mild cognitive impairment	NR
Kipen [20]	Case-control	Australia	60	Cases: 76 Controls: 72	Alzheimer’s disease, all-cause dementia	NR
Shore [40]	Case-control	USA	20	Cases: 76 Controls: 71	Alzheimer’s and non-Alzheimer’s type dementia, mental status	NR
**Secondary hyperparathyroidism**						
Driessen^d^ [37]	Cross-sectional	Germany	59	53	Global cognitive function in MMS	NR
Gilli^d^ [18]	Cross-sectional	Italy	54	51	Cognitive performance in global cognitive function & memory	NR
Leinau^d^ [38]	Cross-sectional	USA	109	61	Global cognitive function in MMSE, executive dysfunction	NR
Jorde^a^ [41]	Case-control	Norway	84	Cases: 62 Controls: 63	Cognitive impairment in memory, attention, processing speed, executive functions, verbal fluency	Age, gender, education, BMI, health score, serum calcium, serum 25-hydroxyvitamin D^e^
**Hypoparathyroidism**						
Aggarwal [23]	Case-control	India	132	Cases: 37 Controls: 37	Cognitive impairment in global score combining 10 cognitive tests for attention, memory, orientation, language, visuo-spatial functioning, executive function, verbal quotient	NR
Kowdley [22]	Case-control	USA	22	55	Cognitive impairment in memory, concentration & attention, perceptual organization, verbal fluency & learning	NR

MMSE: Mini Mental State Examination; CDR: Clinical Dementia Rating scale; MMS: Mini Mental State, NR: Not reported; CKD: chronic kidney disease.

^a:^Study reporting results for more than one type of research design (i.e. prospective/pre-post and cross-sectional or case-control) but basic characteristics are the same, therefore only prospective designs are included here.

^b^: No control group.

^c^: mean age of six groups.

^d^: studies included participants undergoing dialysis.

^e^:unclear whether calcium /vitamin D is treated as a potential covariate or one of the predictors in linear regression models.

### Assessment of parathyroid hormone

Serum PTH levels were biochemically confirmed by radioimmunoassay methods. Pre- and postoperative mean PTH levels were reported in nine studies [9, 10, 12, 13, 15, 16, 31, 32, 35]. Three studies reported only preoperative mean PTH levels [14, 33, 34]. The majority of studies used a PTH-level reference range of 10–69 pg/ml for patients without kidney disease, whereas it varied across the studies with samples of patients on dialysis [10, 18, 36–38]. More specifically, primary hyperparathyroidism was defined based on normal, elevated or high PTH levels but elevated calcium levels and normal serum creatinine levels. Secondary hyperparathyroidism was generally (where reported) determined according to dialysis status, high PTH levels but low/normal calcium levels, and serum creatinine or drugs influencing levels of calcium (e.g. vitamin D analogs). Hypoparathyroidism patients were identified through a diagnosis of hypoparathyroidism documented by low PTH levels and hypocalcaemia [22] or normal serum creatinine and hyperphosphataemia in addition to those markers [23] (exact biomarker levels not reported).

### Assessment of cognitive function and dementia

Forty-eight different tests and subtests were used across studies to assess cognitive function (S2 Table). Ten studies utilized neuropsychological batteries, and the Wechsler Adult Intelligence Scale (WAIS), the Trail making Tests A and B, and the Stroop Color-Word test seemed to be the predominant tests. The rest of the studies used between one and four tests that measured cognitive abilities in specific domains, such as memory, attention and executive function. The only common characteristic across studies is that they all measured some aspect of memory (e.g. visual, verbal or working memory). Screening tests to detect mild cognitive impairment were administered in three studies [10, 16, 19]; two used the Mini Mental State Examination (MMSE) and the Clinical Dementia Rating scale [10, 19]. All-cause dementia and Alzheimer’s disease were confirmed by standardized diagnostic criteria (DSM, NINCDS-ADRDA) and one study used the Dementia Screening Scale of Hasegawa score [21]. Vascular dementia was diagnosed according to the NINDS-AIREN criteria and the guidelines by Erkinjuntti [42].

### Quality assessment

Assessment of methodological quality (see S3 Table) was challenging because the reporting was often incomplete. No study met satisfactorily all the quality indicators from the checklist (reporting, internal and external validity, power) or received a high quality rating. Studies were almost uniformly distributed in the low [14–18, 20, 21, 29, 30, 32, 34–36, 40] and moderate [9, 10, 12, 13, 19, 22, 23, 31, 33, 37–39, 41] quality categories. All but one [15] of the studies were observational. The only randomized control trial retrieved [15] was a small pilot study with an unsuccessful intervention and incomplete reporting of cognitive outcomes, and it does not contribute significantly to the body of evidence. Although study objectives were clearly described in all but three studies [21, 39, 41], the representativeness of the study population was often difficult to determine. Only five studies reported power calculations [9, 12–14, 31] and as study sizes were generally small, it is possible they had insufficient power to detect associations or change in cognitive measures. Potential confounders were poorly described and rarely adjusted for in the analyses (see Table 1). Details of participants lost to follow-up were underreported and complete case analysis appears to be the method used to deal with missing data. Taken together, incomplete reporting, small sample sizes and inadequate adjustment for confounders have potentially compromised the internal and external validity of the included studies.

### Main findings

#### Surgical intervention studies. Primary hyperparathyroidism

Thirteen studies examined the relationship between primary hyperparathyroidism and cognitive function (Table 2). These comprised one randomized controlled trial and twelve pre-post surgery studies (8 with controls and 5 without). The randomized controlled trial incorporated 9 patients in the surgery and control group respectively followed for 6 months [15]. Changes on ten cognitive tests in this small trial were not fully reported. Although a significant ‘change’ in the Stroop Word test was reported at 6 months postoperatively (p = 0.02), it is not clear whether this change was observed for both surgical patients and controls, or whether this change represented improved or poorer cognition in comparison with baseline.

**Table 2:** Main findings of surgical intervention studies relating to parathyroid conditions, cognitive function and dementia.

**Exposure and Study**	**Study design**	**Global cognitive function**	**Memory**	**Executive function**	**Attention**	**Other cognitive domain**	**Dementia**
**Primary hyperparathyroidism**							
Perrier^c^ [15]	RCT		—	-	—?	—	
Chiang^a^ ^,^ ^d^ [14]	Pre-post		—	-	-	—	
Cogan [29]	Pre-post	-	—	—	-	—	
Dotzenrath^a^ ^§^ [16]	Pre-post	↑	↑-	-			
Goyal^a^ [29]	Pre-post	-	-				
Numann^a^ ^,^ ^c^ ^,^ ^d^ [17]	Pre-post		↑ ↑ ↑—	-		↑ ↑ ↑—	
Roman^a^ ^,^ ^c^ ^,^ ^d^ [9]	Pre-post		↑—-				
Walker^a^ ^,^ ^c^ ^,^ ^d^ [12]	Pre-post		↑ ↑ ↑—		↑—	-	
Babinska^a^ [13]	Pre-post		↑ ↑—	-	-	—	
Casella [34]	Pre-post^b^	-					
Mittendorf^a^[33]	Pre-post^b^			↑	↑ ↑		
Prager [32]	Pre-post^b^		↑		↑ ↑—		
Roman [31]	Pre-post^b^		↑ ↑ ↑ ↑ ↑ ↑				
Benge^d^[35]	Pre-post^b^	↑					
**Secondary hyperparathyroidism**		**Global cognitive function**	**Memory**	**Executive function**	**Attention**	**Other cognitive domain**	**Dementia**
Chou^a^[10]	Pre-post	↑					↑
Cogan [29]	Pre-post	-	—	↑ ↑	-	↑ ↑	

Note: Empty cells indicate no relevant results. Arrows and dashes indicate the number of tests conducted under each domain.

^a^ Study reporting results for more than one type of research design (e.g. prospective and cross-sectional, pre-post surgery and case-control, see S1 Table).

^b^: Pre-post surgery study with no control group.

^c^: Total number of tests used or comparisons made not clearly reported.

^d^: P values not fully reported.

↑: Postoperative improvement compared to controls (p <. 05) indicating elevated PTH levels were harmful.

–: No statistically significant association/difference (p <. 05) observed in tests.

?: Direction of change unclear.

Eight of the observational studies of primary hyperparathyroidism compared patients receiving parathyroidectomy to controls. Five out of these eight studies reported significant improvement of surgical patients in comparison with controls on at least one cognitive test [9, 12, 13, 16, 17] although the precise number of tests incorporated was unclear in two studies [9, 17]. The results of statistical significance tests were not fully or adequately reported in five studies [9, 12, 14, 16, 17]. Three studies that measured global cognitive function provide mixed results; two showing no change [29, 34] and one reporting significant postoperative improvement [16]. Memory was assessed in all eight studies. Five studies reported improvement on one to three memory subtests incorporated in their assessments [9, 12, 13, 16, 17] (Table 2). Yet, memory improvement was not universal within a given study which is reflected by the nineteen memory tests/subtests with no postoperative change. None of the five studies that examined executive function observed changes postoperatively [13, 14, 16, 17, 29], whereas attention was improved postoperatively in one [12] out of four studies [12–14, 29]. Other areas with better postoperative performance among the numerous tests utilized across studies included abstract verbal reasoning, visual processing and spatial perception as measured by the WAIS scale [17].

Three out of the five studies pre-post parathyroidectomy without a control group reported significant improvement on at least one cognitive measure [13, 31–33] (Table 2). One study reported no significant change in global cognitive function [34]. Memory performance was improved significantly in two studies [31, 32] and executive function in one study [33]. Postoperative improvement in attention tasks was observed in two studies [32, 33]. Finally, one pre-post study [35] categorized patients having clinically significant impairment if their performance on three or more of fifteen cognitive tests was below a z-score of 1.5. Nearly 40% of patients who met the criteria for clinically significant impairment preoperatively were no longer impaired a month after parathyroidectomy. Information processing speed was the main domain with significant change. This is also the only study reporting decline at follow-up. 18.6% of patients were found impaired postoperatively, mainly on memory and fewer on executive function measures [35].

Despite that these studies [31–35] support a beneficial effect of parathyroidectomy and therefore reduction of PTH levels on cognitive outcomes, the lack of a control group and the short-term follow-up do not allow differentiation between improvements due to surgery and improvements due to practice effects. Taken together, studies of primary hyperparathyroidism provide mixed evidence to support an association with poorer memory and other cognitive domains.

#### Secondary hyperparathyroidism

Both pre-post studies [10, 29] examined the effect of parathyroidectomy on global cognitive function, but only one demonstrated improved performance after surgery [10]. The study also reported improved dementia rating scores (global) measured by the CDR scale following surgery for the study group compared to controls with secondary hyperparathyroidism; memory was the domain with the greatest postoperative improvement [10]. No postoperative difference was observed in tests of memory and attention but parathyroidectomy was associated with improvement in executive function, visual-motor skills and scoring of general intelligence items in the second study [29].

### Non-surgical studies

#### Serum PTH levels

Six studies examined the relationship between serum PTH levels and cognitive function [19, 36, 39, 40] or dementia [19–21, 39, 40] (Table 3). Björkman and colleagues [19] conducted the only prospective study. Participants with elevated PTH levels (≥62ng/L; normal <55 ng/L) had a two-fold increased risk of cognitive decline and all-cause dementia over one and five years [19]. No associations were observed at the 10-year follow-up, though non-random attrition on the basis of baseline PTH levels was observed and the sample size was greatly reduced by that point. This suggests that PTH elevation may precede cognitive decline. However, around 36% of participants already had cognitive impairment at baseline and therefore reverse causation is possible. One cross-sectional and four case-control studies also provide some support for an association between serum PTH and poor cognitive function/impairment or dementia. Higher serum PTH was associated with poorer performance on measures of global cognitive function and executive function in the cross-sectional study [36]. Findings from two case-control studies showed no significant difference in PTH levels between groups of patients with Alzheimer’s disease, other dementias, cognitive impairment and controls [39], or between patients with Alzheimer’s disease and a mixed control group with other type of dementia or a psychiatric illness [40]. Conversely, two additional case-control studies reported significantly higher serum PTH in women with dementia compared to controls [20, 21]. The results of statistical significance tests were not fully or adequately reported in two studies [36, 40]. In summary, mixed findings for both cognitive function and dementia were suggestive of an association with higher PTH levels though equivocal.

**Table 3:** Main findings of non-surgical studies relating to PTH levels, parathyroid conditions, cognitive function and dementia.

**Exposure and Study**	**Study design**	**Global cognitive function**	**Memory**	**Executive function**	**Attention**	**Other cognitive domain**	**Dementia**
**Serum PTH levels**							
Björkman^a^[19]	Prospective	↓					↓
Kalaitzidis^b^ [36]	Cross-sectional	↓		↓			
Johansson [39]	Case-control	-					-
Ogihara [21]	Case-control						↓
Kipen [20]	Case-control						↓-
Shore^b^ [40]	Case-control	-					-
**Secondary hyperparathyroidism**		**Global cognitive function**	**Memory**	**Executive function**	**Attention**	**Other cognitive domain**	**Dementia**
Driessen^c^ [37]	Cross-sectional	↓					
Gilli^c^ [18]	Cross-sectional	↓	↓				
Leinau^c^ [38]	Cross-sectional	-		↓			
Jorde^a^ [41]	Case-control		↓———	- -	↓——	↓	
**Hypoparathyroidism**		**Global cognitive function**	**Memory**	**Executive function**	**Attention**	**Other cognitive domain**	**Dementia**
Aggarwal [23]	Case-control	↓	↓ ↓	↓—	↓ ↓	↓ -	
Kowdley [22]	Case-control		———		——	↓—	

Note: Empty cells indicate no relevant results. Arrows and dashes indicate the number of tests conducted under each domain.

^a^: Study reporting results for more than one type of research design (e.g. prospective and cross-sectional, pre-post surgery and case-control).

^b^: P values not fully reported.

^c^: studies included participants undergoing dialysis

↓: Poorer performance compared to controls or in relation to PTH (p <. 05) indicating abnormal PTH levels are harmful.

–: No statistically significant association/difference (p <. 05) observed in tests.

#### Secondary hyperparathyroidism

Four non-surgical studies examined the relationship between secondary hyperparathyroidism and a number of cognitive domains [18, 37, 38, 41] (Table 3). All but one [41] of the studies were conducted in patients with chronic kidney disease, however this did not appear to be accounted for in the analyses. All three cross-sectional studies [18, 37, 38] in this group assessed global cognitive function; two studies [18, 37] reported that higher mean PTH levels were significantly associated with poorer cognitive performance. Memory was assessed in two studies [18, 41]: the cross-sectional study found worse performance in a memory scale for those with higher PTH levels [18]. The case-control study [41] reported that patients with secondary hyperparathyroidism showed poorer performance in one out of seven tests of memory (i.e. working memory) compared to the age- and sex-matched control group. Patients’ performance was also found to be significantly different in one out of four tests of attention. Another area that appears to be influenced was language. No difference between groups was found in this study in two executive function tests [41]. By contrast, cross-sectional results showed that elevated serum PTH was associated with executive dysfunction in one study [38]. Taken together, evidence from non-surgical studies suggests some degree of cognitive impairment in patients with secondary hyperparathyroidism. No consistent pattern related to specific cognitive domains could be identified in this condition.

#### Hypoparathyroidism

Two case-control studies in hypoparathyroidism [22, 23] provide limited evidence to suggest increased odds of cognitive dysfunction (Table 3). Both studies used multiple tests covering a range of cognitive domains. Patients had poorer performance on at least one test in comparison with matched controls. Verbal fluency test (out of 10 tests in total) was significantly different compared to controls in one of the studies [22]. The second study observed poorer performance for patients across multiple domains including global cognitive function, verbal and visual memory, executive function, attention, and visuospatial skills [23].

### Role of calcium and vitamin D

Calcium was hypothesized to be a potential confounder in one study [19] and a potential mechanism to explain the association between PTH and cognitive dysfunction or dementia in sixteen studies [9, 10, 13, 14, 16, 17, 21, 22, 29, 31, 32, 34, 35, 37, 39, 41]. Only one study adjusted for ionized calcium as a potential confounder though the association between PTH and cognitive decline at one and five years remained [19]. Although three studies [9, 32, 41] reported including calcium levels in regression models, it is unclear whether they treated calcium as a potential confounder or one of the predictors of cognitive function in the statistical models. In a case-control study of hypoparathyroid patients the degree of cognitive dysfunction was related to the degree of intracranial calcification on computed tomography [22]. Postmortem brain calcium levels were also significantly higher in patients with chronic renal failure and secondary hyperparathyroidism in comparison with controls [29]. Vitamin D was proposed to be a potential confounder in two studies [15, 37], and a potential mechanism in seven studies [13, 19–21, 35, 39, 41]. However, no study actually adjusted for serum vitamin D levels as a potential confounder or investigated vitamin D as a potential mechanism in their models. In general the reporting of these important covariates when they were included was inadequate, and their potential role in relation to PTH, cognition and dementia remains largely unclear.

## Discussion

This systematic review synthesized 27 low and moderate quality studies of PTH and parathyroid conditions in relation to cognitive function or dementia. Considerable heterogeneity of study designs, populations, sample size, outcomes and statistical analyses prevented a quantitative synthesis of the results in a meta-analysis. Furthermore, studies were challenging to synthesize due to inadequate reporting. Serum calcium and vitamin D were conceptualized inconsistently as both confounders and causal mechanisms though were not generally incorporated in analyses. No large, well-designed trial has investigated whether normalizing PTH levels is effective at improving cognitive outcomes or reducing the risk of dementia. Findings from observational studies were generally mixed, and offer weak support for a link between PTH and cognitive function or dementia.

The majority of identified studies examined primary hyperparathyroidism and the effect of surgery on cognition (n = 13) providing mixed results for a link with cognitive function. The wide range of cognitive measures and the limited use of global screening tools have contributed to inconsistencies and an irregular pattern of associations. Other than findings of some improvements in various aspects of memory in seven of the thirteen studies in this condition, changes in other cognitive domains—if present- seem to follow a random pattern. Even so, the potential independent role of PTH as opposed to important covariates such as calcium and vitamin D was not systematically investigated. Only two studies [9, 31] provided evidence to support an association between higher PTH levels and poorer cognitive function in primary hyperparathyroidism. Similarly, studies in secondary hyperparathyroidism (where we see high PTH but low calcium and vitamin D levels) showed some evidence for impaired cognitive function. Kidney function, however, was generally not adjusted for in these studies as a potential confounder (e.g. statistical adjustment for glomerular filtration rate in the analysis [19]). Again, the pattern of changes was rather inconsistent and it is based on a small number of studies of observational nature (cross-sectional and case-control) and poor quality [18, 37, 38, 41].

The review also suggests that potential associations with cognition may not be limited to hyperparathyroidism but extent to generally abnormal PTH levels. Thus, low PTH may have a negative influence on cognition as indicated by the two small case-control studies in hypoparathyroidism [22, 23]. Studies of serum PTH levels and cognitive function are limited and evidence from high quality data is lacking. Associations with dementia risk are understudied. Only one long-term prospective study was identified [19] supporting a link between higher PTH levels and increased risk of dementia. Currently, this study [19] along with the surgical intervention studies by Roman and colleagues [9, 31] concentrate the evidence to support a potentially independent role of PTH in cognition and dementia. Certainly more research is needed to confirm this hypothesis. Indeed, PTH excess has been associated with various health outcomes and has the potential to influence cognitive function and dementia risk but the underlying mechanisms to explain such relationships, although suggested, were not actually explored in the reviewed studies (e.g. using mediation analysis).

Several mechanisms have been proposed to explain possible links between PTH, cognitive decline and dementia. Traditionally, impaired cognition in primary hyperparathyroidism has been attributed to hypercalcaemia. High serum calcium levels have been associated with faster decline in cognitive function in the elderly [43]. It is hypothesized that calcium ions cross the blood-brain barrier which may result in calcium overload, neuronal signaling disruption or atrophy in hippocampus and Alzheimer’s disease [2]. Calcium deposits in the brain have also been related to frontal-subcortical dementia [44]. Furthermore, PTH is related to vitamin D levels and as both regulate calcium levels, they are linked through a feedback loop; decreasing serum vitamin D levels are associated with increasing PTH levels. Long-term vitamin D deficiency may induce secondary hyperparathyroidism and is also associated with increased risk of cognitive decline and Alzheimer’s disease [45]. A recent meta-analysis indicated that higher PTH levels were associated with nearly 50% increased risk of cardiovascular disease events [46]. Arterial stiffness and endothelial dysfunction have been observed in patients with primary hyperparathyroidism and may result in cognitive impairment [47]. PTH crosses the blood-brain barrier and PTH excess in animal models has been linked to enhanced release of vasopressin and vasoconstriction [48]. Additionally, hypoperfusion in several cortical regions involved in memory and learning has been observed in patients with primary hyperparathyroidism compared to controls [3]. Normalization of PTH levels may influence brain metabolism and may be responsible for the postoperative improvement in cognition demonstrated in patients with primary hyperparathyroidism. While a range of different mechanisms have been proposed their possible clinical relevance is unclear and remains to be established.

Our analysis is the first systematic review to examine associations between PTH, cognition and dementia, covering the whole spectrum of parathyroid conditions and also serum PTH levels. The review was based on a comprehensive search strategy without date or language restrictions, which supports our confidence in identifying all relevant studies. Whilst the broad scope of the review provides a comprehensive picture of current research in this area, it has introduced some heterogeneity. Additionally, owing to the observational nature of all but one of the studies the issue of causality cannot be assessed. Only one randomized controlled trial has been conducted of limited sample size and follow-up. The majority of studies incorporated small sample sizes and are likely to have lacked statistical power to detect associations or significant change. Multiple testing was an issue as many studies incorporated a large number of cognitive tests. Given the low to moderate quality and generally inadequate reporting, it is not possible to draw firm conclusions about the relationship between PTH and cognitive function or dementia. The range of cognitive outcomes, inadequate reporting and methodological heterogeneity meant that it was not possible to conduct a meta-analysis which would have made it easier to estimate if there was a significant pooled association across studies.

## Conclusions

In conclusion, there is currently no basis to suggest a causal relationship between PTH and cognitive function or dementia. Mixed findings from the available studies are supported by low and moderate quality data susceptible to confounding effects and limited external validity. To date, no trial has examined this relationship where PTH levels were successfully normalized in a large population at risk of dementia. Given the plausible mechanisms to suggest abnormal PTH levels may lead to cognitive dysfunction and an increased risk of dementia, further investigation is warranted. Large well-designed prospective studies would be useful and could also investigate related factors including calcium and vitamin D which have been conceptualized as both confounders and causal mechanisms in the previous literature.

## Supporting Information

S1 Table Summary of main findings relating to parathyroid hormone, cognitive function and dementia. (DOCX)

S2 Table Cognitive domains and tests used to assess cognitive function or dementia in included studies. (DOCX)

S3 Table Quality assessment of included studies. (DOCX)

S1 Text Systematic review protocol. (DOCX)

S2 Text PRISMA checklist. (DOC)
