# Supplementary material for: Parathyroid Hormone, Cognitive Function and Dementia: A Systematic Review
Source: PLoS One. 2015 May 26;10(5):e0127574. doi: 10.1371/journal.pone.0127574 (PMC4444118; doi:10.1371/journal.pone.0127574)
Supplement: S1 Table — (DOCX) [file pone.0127574.s001.docx]

**Supporting information file 1**

**S1 Table.** Summary of main findings relating to parathyroid hormone, cognitive function and dementia

| **Exposure and study** | **Design** | **Global cognitive function** | **Memory** | **Executive function** | **Attention** | **Other cognitive domain** | **Dementia** |
| --- | --- | --- | --- | --- | --- | --- | --- |
| **Surgical intervention studies** |  |  |  |  |  |  |  |
| **Primary hyperparathyroidism** |  | **Global cognitive function** | **Memory** | **Executive function** | **Attention** | **Other cognitive domain** | **Dementia** |
| Perrier^c^ [15] | RCT |  | - - | - | - - - ? | - - - |  |
| Chiang^a,d^ [14] | Pre-post  Case-control |  | - - - -  - - - - | -  - | -  - | - -  - - |  |
| Cogan [28] | Pre-post | - | - - | - - | - | - - |  |
| Dotzenrath^a,d^ [16] | Pre-post  Case-control | ↑ | ↑  ↓ | -  ↓ |  | -  - |  |
| Goyal^a^ [29] | Pre-post  Case-control |  | -  - |  |  | -  - |  |
| Numann^a,c,d^ [17] | Pre-post  Case-control |  | ↑ ↑ ↑ - - - - - -  - - - - - - | -  ↑ |  | ↑ ↑ ↑ - - -  - - - |  |
| Roman^a,c,d^ [9] | Pre-post  Case-control |  | ↑ - -  - - - |  |  |  |  |
| Walker^a,d^ [12] | Pre-post  Case-control |  | ↑ ↑ ↑ - -  ↓ ↓ ↓ - - |  | ↑ - - -  - - - - | -  ↓ |  |
| Babinska^a^ [13] | Pre-post  Case-control |  | ↑ ↑ - - - - - - - - -  ↓ ↓ ↓ ↓ ↓ ↓ ↓ ↓ - - - | -  - | -  ↓ | - - -  ↓ ↓ - |  |
| Casella [33] | Pre-post^b^ | - |  |  |  |  |  |
| Mittendorf^a^[32] | Pre-post^b^  Cross-sectional |  |  | ↑  - | ↑ ↑  - - |  |  |
| Prager [31] | Pre-post^b^ |  | ↑ |  | - - - - |  |  |
| Roman [30] | Pre-post^b^ |  | ↑ ↑ ↑ ↑ ↑ ↑ |  |  |  |  |
| Benge^d^[27] | Pre-post^b^  Cross-sectional | ↑  ↓ |  |  |  |  |  |
| **Secondary hyperparathyroidism** |  | **Global cognitive function** | **Memory** | **Executive function** | **Attention** | **Other cognitive domain** | **Dementia** |
| Chou^a^[10] | Pre-post  Case-control | ↑  ↓ |  |  |  |  | ↑  ↓ |
| Cogan [28] | Pre-post | - | - - | ↑ ↑ | - | ↑ ↑ |  |
| **Non-surgical studies** |  |  |  |  |  |  |  |
| **Serum PTH levels** |  |  |  |  |  |  |  |
| Björkman^a^[19] | Prospective  Cross-sectional | ↓  ↓ |  |  |  |  | ↓  ↓ |
| Kalaitzidis^d^ [34] | Cross-sectional | ↓ |  | ↓ |  |  |  |
| Johansson [37] | Case-control | - |  |  |  |  | - |
| Ogihara [21] | Case-control |  |  |  |  |  | ↓ |
| Kipen [20] | Case-control |  |  |  |  |  | ↓ - |
| Shore^d^ [38] | Case-control | - |  |  |  |  | - |
| **Secondary hyperparathyroidism** |  |  |  |  |  |  |  |
| Driessen [35] | Cross-sectional | ↓ |  |  |  |  |  |
| Gilli [18] | Cross-sectional |  | ↓ |  |  | ↓ |  |
| Leinau [36] | Cross-sectional | - |  | ↓ |  |  |  |
| Jorde^a^[41] | Case-control |  | ↓ - - - - - | - - | ↓ - - - | ↓ - |  |
| **Hypoparathyroidism** |  |  |  |  |  |  |  |
| Aggarwal [39] | Case-control | ↓ | ↓ ↓ | ↓ - - | ↓ ↓ | ↓ - |  |
| Kowdley [40] | Case-control |  | - - - - - |  | - - - - | ↓ - - - |  |

Note: Empty cells indicate no relevant results. Arrows and dashes indicate the number of tests conducted under each domain.

^a^: Study reporting results for more than one type of research design (e.g. prospective and cross-sectional, pre-post surgery and case-control).

^b^: Pre-post surgery study with no control group.

^c^: Total number of tests used or comparisons made not clearly reported.

^d^: P values not fully reported.

↓: Poorer performance compared to controls or in relation to PTH levels.

–: No statistically significant association/difference (p < .05) observed in tests.

↑: Postoperative improvement or better performance at baseline compared to controls (p < .05).

? : Direction of change unclear
