# Supplementary material for: Parathyroid Hormone, Cognitive Function and Dementia: A Systematic Review
Source: PLoS One. 2015 May 26;10(5):e0127574. doi: 10.1371/journal.pone.0127574 (PMC4444118; doi:10.1371/journal.pone.0127574)
Supplement: S2 Table — (DOCX) [file pone.0127574.s002.docx]

**Supporting information file 2**

**S2 Table.** Cognitive domains and tests used to assess cognitive function or dementia in included studies

| **Exposure and Study** | **Study design/population** | **Number of tests (or subtests)** | **Cognitive domain and tests/criteria^*^** |
| --- | --- | --- | --- |
| **Surgical intervention studies** | | | |
| **Primary hyperparathyroidism** |  |  |  |
| Perrier [15] | RCT | 10^‡^ | Memory, attention, concentration, psychomotor speed, and dexterity: Digit Span and Digit Symbol subtests of WAIS, Controlled Oral Word Association test, Grooved Pegboard test, Trail Making test A and B, Hopkins Verbal Learning Test Revised, PASAT, and the **Stroop** Color and **Word** test |
| Chiang [14] | Pre-post & Case-control | 8 | Visual memory: Unusual Shapes Test; Verbal memory: Royal Melbourne Memory for Prose Test (3 subtests); Attention: Digit Symbol subtest of WAIS; Executive function: Stroop Color-Word test; Verbal and visual recognition (2 subtests) |
| Cogan [28] | Pre-post | 8 | General cognitive function, nonverbal problem solving, and visual spatial skills: Raven’s Progressive Matrices, Neurologic Index of Mental Impairment, Visual; Motor Items-VMI; Memory: WMS; Visual memory: memory for Designs test; Attention & processing speed: Digit Symbol; Visuospatial skills & executive function: Trail Making Tests A and B |
| Dotzenrath [16] | Pre-post & Case-control | 4 | Cognitive impairment: **DEM Tect**; Visual memory: Benton Visual Retention Test; Verbal memory: Multiple Word Test ; Visuospatial skills & executive function: ZVT |
| Goyal [29] | Pre-post & Case-control | 2 | Memory, learning, executive functions & global cognitive function: Memory Scale, and Intellectual Process Scale for Hindi-knowing |
| Numann [17] | Pre-post & Case-control | 16^‡^ | Battery to test memory functioning, attention and concentration skills, fine motor coordination, verbal abilities, visuospatial functions, planning and organizational skills: **WMS Form** I (6 subtests), Information, Vocabulary, **Similarities & Block Design subtests of WAIS**, Facial Recognition Test, **Benton Visual Retention Test** (4 subtests), Trail Making tests A and B |
| Roman [9] | Pre-post & Case-control | 3^‡^ | Verbal memory & learning: Rey’s Auditory Verbal Learning Test; Spatial working memory & visuomotor speed: **Groton Maze Learning Test** (2 subtests) |
| Walker [12] | Pre-post & Case-control | 10 | Visual memory: **Rey’s Visual Design Learning Test**; Verbal memory & learning: Buschke Selective Reminding Test, **WMS Logical Memory Test** (2 subtests); Visual concentration/attention: **Digit Symbol**, Rosen Target Detection Test (2 subtests), Digit SpanNonverbal abstraction: **Booklet Category Test** |
| Babinska [13] | Pre-post & Case-control | 16 | Battery of tests to measure visual, working, and verbal memory, attention, psychometric speed, learning ability, executive functions, intellectual flexibility and planning, verbal fluency: **Benton Visual Retention Test** (2 subtests), **Memory Verbal Learning Test** (DCS-8 subtests), Rey’s Auditory Verbal Learning Test, **Trail Making** Tests **A** and B, Verbal Fluency Test (3 subtests) |
| Casella [33] | Pre-post (no control group) | 1 | General cognitive function (memory, attention, language, calculus, space-time orientation): MMSE |
| Mittendorf [32] | Pre-post (no control group) & Cross-sectional | 3 | Attention & processing speed: **Stroop Word and Stroop Color subtests** Executive function: **Stroop Color-Word subtest** |
| Prager [31] | Pre-post (no control group) | 5 | Short-term memory: **Numbers Memorizing**- part of the Wilde Intelligence Test; Attention & concentration: **d2-Test of Attention** (4 subtests) |
| Roman [30] | Pre-post (no control group) | 6 | Verbal memory & learning: **Rey’s Auditory Verbal Learning Test** (2 subtests) ; Spatial working memory & error monitoring: **Groton Maze Learning Test** (4 subtests) |
| Benge [27] | Cross-sectional | 15 | Verbal memory and learning: Hopkins Verbal Learning Test Revised (3 subtests); Attention: Digit Span; Processing speed: PASAT (2 trials), Trail Making test A, Digit Symbol, Stroop Color, Stroop Word tests; Executive function: Trail Making Test B, Controlled Oral Word Association, Stroop Color-Word test; Motor speed & dexterity: Grooved Pegboard tests (2 subtests) |
| **Secondary hyperparathyroidism** |  |  |  |
| Chou [10] | Pre-post & Case-control/ SHPT dialysis | 2 | General cognitive function/decline(memory, attention, language, calculus, space-time orientation, judgment, problem solving): **MMSE**, **CDR scale** |
| Cogan [28] | Pre-post (SHPT) | 8 | General cognitive function, nonverbal problem solving, and visual spatial skills: **Raven’s Progressive Matrices**, Neurologic Index of Mental Impairment, Visual Motor Items-**VMI**; Memory: WMS; Visual memory: memory for Designs test; Attention & processing speed: Digit Symbol; Visuospatial skills & executive function: **Trail Making Tests A and B** |
| **Non-surgical studies** | | | |
| **Serum PTH levels** |  |  |  |
| Björkman[19] | Prospective & cross-sectional | 2 | Cognitive function/impairment: **MMSE**; Dementia: **CDR** scale |
| Kalaitzidis[34] | Cross-sectional | 2 | Cognitive function: **MMSE**; Visual and executive function: **Clock-drawing test** |
| Johansson[37] | Cross-sectional | 1 | Cognitive impairment: MMSE; Dementia: DSM-IV, NINDS-AIREN, guidelines by Erkinjuntti et al^§^ |
| Ogihara [21] | Cross-sectional & case-control | 1 | Cognitive function, Alzheimer’s disease, Vascular dementia: **Dementia Screening Scale of Hasegawa** |
| Kipen [20] | Case-control | 2 | Dementia: **DSM-III-R**; Alzheimer’s disease: NINDS-ADRDA |
| Shore [38] | Case-control | 2 | Alzheimer’s disease, non- Alzheimer’s type dementia: DSM-III, Mental Status Questionnaire |
| **Secondary hyperparathyroidism** |  |  |  |
| Driessen [35] | Cross-sectional/ SHPT dialysis | 1 | General cognitive function (memory, attention, language, orientation): Mini Mental State |
| Gilli [18] | Cross-sectional/ SHPT dialysis | 2 | Memory: **WMS scale**; Global cognitive function & reasoning ability: **WAIS** scale |
| Leinau [36] | Cross-sectional/ SHPT dialysis | 2 | General cognitive function (memory, attention, language, orientation): MMSE ; Executive dysfunction: **EXIT25** |
| Jorde [41] | Cross-sectional & case-control | 14 | Working memory: **Digit Span forward**, Digit Span backwards, Seashore Rhythm test; Verbal memory: delayed recall from WMS-R, verbal recall subtest from the California Verbal Learning Test, Vocabulary subtest from WAIS; Attention & processing speed: **Digit Symbol**, the California Computerized Assessment Package, **Stroop Color, Stroop Word**, Trail Making A; Word fluency: Controlled Oral Word Association test; Cognitive flexibility/ executive function: Trail Making B, Stroop Color-Word |
| **Hypoparathyroidism** |  |  |  |
| Aggarwal [39] | Cross-sectional | 10 | Global cognitive score based on 10 tests for:; Memory, language, orientation: Hindi Mental State Examination; Visual attention and task switching: **Trail Making Tests A** **and B** (2 subtests); Verbal and non-verbal memory: **PGI-Memory scale**; Visuospatial gestalt and micrographia: **Bender Gestalt Test**; Information, comprehension and verbal intelligence: Verbal Adults Intelligence Scale (VAIS); Perception, memory, constructive abilities: **Benton Visual Retention Test** (BVRT); Executive functions, response inhibition, cognitive flexibility: Stroop Colour and **Stroop Word** Test (3 subtests) |
| Kowdley [40] | Case-control | 13 | Visual memory: Benton Visual Retention Test (2 subtests); Verbal memory & learning: California Verbal Learning Test (2 subtests), Serial Digit Learning Test, Vocabulary subtest from WAIS; Concentration & attention: Digit Span tests, Trail Making Tests A and B; Verbal fluency: **Controlled Oral Fluency Test**; Perceptual organization: Picture Completion and Block Designs of WAIS-R |

PTH: parathyroid hormone, SHPT: secondary hyperparathyroidism, PASAT: Paced Auditory Serial Addition Task MMSE: Mini Mental State Examination, CDR: Clinical

Dementia Rating scale, DSM: Diagnostic and Statistical Manual of Mental Disorders/Revised, NINDS-AIREN: National Institute of Neurological Disorders and Stroke

and Association Internationale pour la Recherché et l'Enseignement en Neurosciences, NINDS-ADRDA: National Institute of Neurological and Communicative Disorders

and Stroke- Alzheimer’s Disease and Related Disorders Association criteria , WMS: Wechsler Memory Scale, WAIS-R: Wechsler Adult Intelligence Scale-Revised,

EXIT25: The Executive Interview

Bold font represents tests with significant associations with PTH levels in cross-sectional studies or postoperative improvement in pre-post studies

^*^:tests are categorized under cognitive domains according to the description provided by the relevant study

‡:total number of tests used or comparisons made not clearly reported

§: Erkinjuntti T, Inzitari D, Pantoni L, Wallin A, Scheltens P et al. Research criteria for subcortical vascular dementia in clinical trials. Journal of Neural Transm 2000; Suppl 59: 23-30.
