# Supplementary material for: Parathyroid Hormone, Cognitive Function and Dementia: A Systematic Review
Source: PLoS One. 2015 May 26;10(5):e0127574. doi: 10.1371/journal.pone.0127574 (PMC4444118; doi:10.1371/journal.pone.0127574)
Supplement: S3 Table — (DOCX) [file pone.0127574.s003.docx]

**S3 Table.** Quality assessment of included studies

|  | **Quality subscales from checklist** | | | | |  |
| --- | --- | --- | --- | --- | --- | --- |
| **Study** | **Reporting** | **External validity** | **Internal validity-bias** | **Internal validity-confounding (selection bias)** | **Power** | **Overall Quality** |
| **Surgical intervention studies** | | | | | | |
| Perrier[15] | moderate | low | moderate | low | low | Low |
| Chiang[14] | moderate | low | low | low | low | Low |
| Cogan[28] | moderate | low | low | low | low | Low |
| Dotzenrath[16] | moderate | low | low | low | low | Low |
| Goyal[29] | low | moderate | moderate | moderate | low | Low |
| Numann[17] | low | low | moderate | low | low | Low |
| Roman[9] | moderate | moderate | moderate | moderate | good | Moderate |
| Walker[12] | moderate | moderate | moderate | moderate | good | Moderate |
| Babinska[13] | moderate | low | moderate | low | good | Moderate |
| Casella[33] | low | low | low | low | low | Low |
| Mittendorf[32] | moderate | moderate | good | good | low | Moderate |
| Prager[31] | moderate | low | moderate | good | low | Low |
| Roman[30] | good | low | moderate | moderate | good | Moderate |
| Benge[27] | low | low | moderate | good | low | Low |
| Chou[10] | good | low | good | low | low | Moderate |
| **Non-surgical studies** | | | | | | |
| Björkman[19] | moderate | moderate | good | moderate | low | Moderate |
| Kalaitzidis[34] | moderate | low | good | low | low | Low |
| Jorde[41] | moderate | moderate | moderate | moderate | low | Moderate |
| Ogihara[21] | moderate | low | moderate | low | low | Low |
| Johansson[37] | moderate | good | good | low | low | Moderate |
| Kipen[20] | good | low | low | low | low | Low |
| Shore[38] | low | low | low | moderate | low | Low |
| Chou[10] | good | low | good | low | low | Moderate |
| Driessen[35] | good | low | good | low | low | Moderate |
| Gilli[18] | moderate | low | moderate | low | low | Low |
| Leinau[36] | moderate | low | good | low | low | Moderate |
| Aggarwal[39] | good | good | good | low | low | Moderate |
| Kowdley[40] | good | moderate | good | good | low | Moderate |
|  |  |  |  |  |  | Low:14 Moderate:13 |
