# Supplementary material for: Parathyroid Hormone, Cognitive Function and Dementia: A Systematic Review
Source: PLoS One. 2015 May 26;10(5):e0127574. doi: 10.1371/journal.pone.0127574 (PMC4444118; doi:10.1371/journal.pone.0127574)
Supplement: S1 Text — (DOCX) [file pone.0127574.s004.docx]

**S1 Text (Protocol)**

**Parathyroid hormone, cognitive function and dementia: systematic review**

**Rationale**

Parathyroid hormone (PTH) is a circulating hormone produced in the parathyroid glands with the major biological function to maintain the extracellular calcium and phosphate levels within normal limits, primarily by targeting cells in bone and kidney [1]. Hypocalcaemia and hyperphosphataemia trigger the secretion of PTH which then helps to restore normal mineral concentrations. This is achieved by stimulating three main processes: i) stimulation of osteoclast resorption to release bone calcium into the circulation, ii) promotion of tubular reabsorption of ionized calcium (Ca^2+^) in the kidneys and excretion of phosphate ions in urine, and iii) increased activity of renal 1-hydroxylase which results in the production of the active form of vitamin D, 1,25-dehydroxyvitamin D (1,25-OHD). Vitamin D induces synthesis of a calcium-binding protein in the intestines that facilitate calcium absorption into the blood [2].

In parathyroid disorders, the balance of this regulatory system is disrupted by changes in the secretion and activity of parathyroid hormone. A state of high PTH levels can cause hyperparathyroidism whereas reduced activity can result in hypoparathyroidism. Traditionally, hyperparathyroidism has been classified as *primary* or *secondary* depending on the origin of excessive secretion of parathyroid hormone. In primary hyperparathyroidism (PHPT), one or more of the four parathyroid glands become overactive and the increase of PTH results in hypercalcaemia. Secondary hyperparathyroidism (SHPT) occurs when one or more components of the calcium regulatory system fail and calcium in the blood is below normal range. Chronic kidney disease, vitamin D deficiency and/ or low calcium intake may cause increased PTH secretion in an attempt to restore calcium availability [3].

Primary hyperparathyroidism (PHPT) is one of the most common endocrine disorders with an estimated prevalence of 2% in postmenopausal women and an age-related elevation in PTH concentrations has been observed [4]. The classical symptoms of PHPT include renal stones, bone disease and gastrointestinal complaints, but are seen less often today mainly due to routine biochemical screening. Parathyroidectomy, the surgical removal of one or more parathyroid adenomas, is the only curative treatment for PHPT in symptomatic patients. However, approximately 80% of patients have no obvious signs or symptoms (asymptomatic) and present with more vague symptoms such as weakness and fatigue or report only neurobehavioural symptoms including cognitive dysfunction, irritability, depressive or anxiety symptoms and sleep disorders.

Hyperparathyroidism, mainly PHPT and secondary hyperparathyroidism (SHPT) caused by chronic kidney disease, has been associated with a range of impaired health outcomes including increased fracture risk, cardiovascular mortality, psychiatric morbidity, cognitive dysfunction and dementia [5-8]. Numerous case-control studies have assessed neuropsychological or cognitive functioning in PHPT patients pre- and postoperatively. Findings provide some evidence for impairment in various cognitive domains including verbal memory, executive function and concentration in PHPT patients before parathyroidectomy and subsequent improvement after successful surgery [9-12]. A review examining neuropsychological testing and health-related quality of life in PHPT patients pre- and postoperatively concluded that cognitive tools had been underused and findings for cognitive functioning were ambiguous as only a small number of studies was available [13]. However, more studies have been published since then and a few of them have also examined associations between cognitive changes and serum calcium or PTH levels with mixed findings [10,14].

A relationship between SHPT and cognitive dysfunction, namely improved MMSE scores after parathyroidectomy, has also been observed [15]. Recently, prospective studies have investigated high parathyroid hormone levels independent of PHPT or SHPT related to kidney failure in relation to cognitive functions. Findings suggested people with high serum PTH concentrations were more likely to score lower in cognitive tests [16] and show greater cognitive decline over a 5-year period [17].

Hypoparathyroidism is characterized by PTH insufficiency or absence. It may be the result of different conditions such as autoimmune destruction or genetic defects in PTH biosynthesis, secretion and parathyroid gland development, but it is most commonly observed after neck surgery due to removal of or damage to the glands. Hypoparathyroidism is associated with low serum calcium levels, frequently leading to hypocalcaemia, and reduced 1,25-OHD concentrations[18]. The standard therapeutic approach for hypoparathyroidism is oral calcium and vitamin D supplementation, but treatment is often challenging.

Patients with hypoparathyroidism often complain of general muscle cramps, numbness around the mouth, chronic headaches, insomnia, fatigue and ‘brain fog’. Older documentations have reported associations with psychotic manifestations, delirium and dementia [19]. Cognitive deficits have also been associated with hypoparathyroidism. A study of 11 patients with hypoparathyroidism revealed that there were significant differences in the neuropsychological tests compared to controls and 65% of the patients were cognitively impaired suggesting a link to cerebral calcification [20]. However, other than a few isolated case reports over the past two decades, cognitive symptoms in hypoparathyroidism have been less researched than its counterpart condition.

Given that increases in PTH concentrations are age-related, successful management of serum PTH may be an additional useful tool for the prevention of cognitive decline or dementia in older populations. Hypoparathyroidism affect calcium levels in the blood with a wide spectrum of symptoms and is potentially associated with cognitive impairment too. However, to date, evidence on the prognostic value of altered parathyroid hormone levels in cognitive function and dementia have not yet been synthesized and evaluated systematically.

**Purpose**

There is evidence to suggest inappropriately low or high serum PTH levels are associated with increased risk for a range of adverse health outcomes including cognitive impairment. The purpose of this review is to investigate and evaluate the potential association between altered serum parathyroid hormone and cognitive function or dementia.

**Review questions**

- Is parathyroid hormone associated with cognitive function or dementia?
- What is the evidence for an independent role of PTH in the associations?

**Population**

- Adults (≥18yrs)

**Exposure/Intervention**

- Serum PTH levels, or
- Diagnosed hypoparathyroidism and hyperparathyroidism (primary/secondary/tertiary), or
- Parathyroidectomy

**Comparators/controls**

Serum PTH levels within normal range, postsurgical PTH levels or a separate control group in trials

**Outcomes to be examined**

If possible, outcome measures will include:

- Cognitive function (including global cognitive function scores, tests and measures of performance on individual cognitive domains, MCI)
- Prevalent and/or incident dementia (including Alzheimer’s disease, vascular dementia and all-cause dementia)

**Methods of synthesis of evidence of association**

The systematic review will synthesize evidence for the potential association between parathyroid hormone and cognitive function(s) or dementia. The effect of parathyroidectomy (to treat hyperparathyroidism) on changes in cognitive function(s) will be examined too. The review will be undertaken following the general principles published by the NHS Centre for Reviews and Dissemination [21].

**Search strategy**

Refer to Appendix 1 for the search strategy for Medline.

The search strategy will comprise the following main elements:

- Searching the following electronic databases: Medline, EMBASE and PsycINFO using the Ovid interface, HMCI, CINAHL, Cochrane Library and Web of Science (up to September 2012)
- Scrutiny of reference lists of included studies
- Hand searching of relevant journals, e.g. Surgery, the Journal of Clinical Endocrinology & Metabolism
- Selected citation searching (‘forward citation chasing’: searching for relevant studies from papers which have cited the studies that meet the inclusion criteria for the review)
- Internet searching of the following relevant websites: Alzheimer’s Society, Alzheimer’s Research UK, Alzheimer’s Association and Hypoparathyroidism UK
- Contact with experts in the field

**Study selection criteria and procedures**

**Types of study to be included**

- Studies that examine the association between PTH and cognitive function(s) or dementia measured by neuropsychological test(s)
- Studies in which cognitive function(s) or dementia and the potential relationship with serum PTH were evaluated by neuropsychological test(s), before and following parathyroidectomy for PHPT or SHPT
- Randomized clinical trials will be included, but it is anticipated their number will be limited if not minimal. Relevant cross sectional and longitudinal studies will be included in the review

**Types of study to be excluded**

- Case-reports
- Studies limited to self-reported measurement of cognitive function
- Narrative reviews, letters, editorials, opinions
- Reports published as meeting abstracts only, where insufficient methodological details are reported to allow critical appraisal of study quality
- Animal models

**Study selection**

The titles and abstracts of references retrieved by the electronic searches will be screened for relevance by one reviewer and independently checked by a second using the pre-specified inclusion/exclusion criteria. Full-text copies of potentially relevant studies will be obtained. Using the same methods, the retrieved articles will be assessed for inclusion. Discrepancies will be resolved by discussion, with involvement of a third reviewer, where necessary. Duplicate papers will be double checked and excluded.

**Quality assessment strategy**

The quality of individual studies will be assessed by one reviewer, and checked by a second reviewer. Any disagreement will be resolved by consensus and if necessary a third reviewer will arbitrate. Appropriate quality assessment criteria will be used depending on the design and reporting of the included studies using a widely used checklist [22].

**Data extraction strategy**

Data will be extracted from included studies by one reviewer into a piloted data extraction form and checked by another reviewer. Discrepancies will be resolved by discussion, with the involvement of a third reviewer if necessary.

**Data synthesis**

Data will be tabulated and discussed in a narrative review. Where appropriate, meta-analysis will be employed to estimate summary measures of effect on relevant outcomes.

If meta-analysis is conducted it will be carried out using fixed and random effects models, using STATA. Heterogeneity will be explored through consideration of the study populations, methods and interventions, by visualisation of results and, in statistical terms, by the χ^2^ test for homogeneity and I^2^ statistic and, where appropriate, using meta-regression. Small study effects (including publication bias) will be visually assessed using funnel plots and quantified using Egger’s statistic.

**Protocol Appendix 1**

Search Strategy example shown for Medline, searched 08.2012

--------------------------------------------------------------------------------

1 parathyroid*.ti,ab. (35714)

2 hyperparathyroidism.ti,ab. (16933)

3 hypoparathyroidism.ti,ab. (3583)

4 'hyper parathyroidism'.ti,ab. (35)

5 'hypo parathyroidism'.ti,ab. (8)

6 parathormon*.ti,ab. (1995)

7 1 or 2 or 3 or 4 or 5 or 6 (46120)

8 (cognitive adj2 (decline or impairment or performance or function* or defect* or deficit* or abilit* or disorder* or assessment*)).mp. [mp=title, abstract, original title, name of substance word, subject heading word, protocol supplementary concept, rare disease supplementary concept, unique identifier] (74581)

9 cognition disorders/ or exp mild cognitive impairment/ (43863)

10 exp Memory/ (88338)

11 (neuropsychological adj (test* or assessment* or evaluation* or symptom*)).ti,ab. (11793)

12 neuropsychological.mp. [mp=title, abstract, original title, name of substance word, subject heading word, protocol supplementary concept, rare disease supplementary concept, unique identifier] (68773)

13 exp neuropsychological tests/ or exp psychometrics/ (104283)

14 8 or 9 or 10 or 11 or 12 or 13 (247281)

15 exp Dementia/ (106793)

16 dement*.ti,ab. (58027)

17 ((vascular or frontotemporal or multi-infarct or 'Lewy Bod*') adj dementia).ti,ab. (6991)

18 Alzheimer*.ti,ab. (73696)

19 15 or 16 or 17 or 18 (139250)

20 14 or 19 (352582)

21 7 and 20 (258)

References-protocol

1. Murray TM, Rao LG, Divieti P, Bringhurst FR. Parathyroid hormone secretion and action: evidence for discrete receptors for the carboxyl-terminal region and related biological actions of carboxyl- terminal ligands. Endocr Rev. 2005;26:178-113.
2. Fraser WD. Hyperparathyroidism. Lancet. 2009;374:145-158.
3. Saleh F, Jorde R, Sundsfjord J, Haug E, Figenschau Y. Causes of secondary hyperparathyroidism in a healthy population: the Tromsø study. J Bone Miner Metab.2006;24:58-64.
4. Marcocci C, Cianferotti L, Silverberg SJ, Bilezikian BJ. Primary hyperparathyroidism in men. In Osteoporosis in men: the effects of gender on skeletal health, 2^nd^ ed. pp.465-478. Elsevier Inc; 2010.
5. Vestergaard P, Mollerup CL, Froksaer VG, Christiansen P, Blichert-Toft M, Mosekilde L. Cohort study of risk of fracture before and after surgery for primary hyperparathyroidism. BMJ 2000; 321:598-602.
6. Andersson P, Rydberg E, Willenheimer R. Primary hyperparathyroidism and heart disease-a review. European Heart Journal 2004; 25:1776-1787.
7. Roman SA, Sosa JA, Mayes L, Desmond E, Boudourakis L, Lin R, Snyder PJ, Holt E, Udelsman R. Parathyroidectomy improves neurocognitive deficits in patients with primary hyperparathyroidism. Surgery 2005; 138: 1121-1129.
8. Papageorgiou SG, Christou Y, Kontaxis T, Bonakis A, Anagnostouli M, Potagas C, Kalfakis N. Dementia as presenting symptom of primary hyperparathyroidism: favourable outcome after surgery. Clin Neurol Neurosurg. 2008;110:1038-1040.
9. Benge JF, Perrier ND, Massman PJ, Meyers CA, Kayl AE, Wefel JS. Cognitive and affective sequelae of primary hyperparathyroidism and early response to parathyroidectomy. J Int Neuropsychol Soc. 2009;15:1002-1011.
10. Roman SA, Sosa JA, Pietrzak RH, Snyder PJ, Thomas DC, Udelsman R, Mayes L. The effects of serum calcium and parathyroid hormone changes on psychological and cognitive function in patients undergoing parathyroidectomy for primary hyperparathyroidism. Ann Surg 2011; 253: 131-137.
11. Mittendorf EA, Wefel JS, Meyes CA, Doherty D, Shapiro SE, Lee JE, Evans DB, Perrier ND. Improvement of sleep disturbance and neurocognitive function after parathyroidectomy in patients with primary hyperparathyroidism. Endocr Pract. 2007: 13:338-344.
12. Prager G, Kalaschek A, Kaczirek K, Passler C, Scheuba C, Sonneck G, Niederle B. Parathyroidectomy improves concentration and retentiveness in patients with primary hyperparathyroidism. Surgery 2002: 132:930-936.
13. Coker LH, Rorie K, Cantley L, Kirkland K, Stump D, Burbank N, Tembreull T, Williamson J, Perier, N. Primary hyperparathyroidism, cognition and health-related quality of life. Ann Surg 2005; 242:642-650.
14. Walker MD, McMahon DJ, Inabnet WB, Lazar RM, Brown I, Vardy S, Cosman F, Silverberg SJ. Neuropsychological features in primary hyperparathyroidism: a prospective study. J Clin Endocrinol Metab.2009; 94:1951-1958.
15. Chou FF, Chen JB, Hsieh KC, Liou CW. Cognitive changes after parathyroidectomy in patients with secondary hyperparathyroidism. Surgery 2008;143: 526-532.
16. Jorde R, Waterloo K, Saleh F, Haug E, Svartberg J. Neuropsychological function in relation to serum parathyroid hormone and serum 25-hydroxyvitamin D levels. The Tromsø study. J Neurol. 2006; 253:464-470.
17. Bjorkman MS, Sorva AJ, Tilvis RS. Does elevated parathyroid hormone concentration predict cognitive decline in older people? Aging Clin Exp Res. 2010; 22:164-169.
18. Bilezikian JP, Khan A, Potts JT, Brandi ML, Clarke B, Shoback D et al. Hypoparathyroidism in the adult: Epidemiology, diagnosis, pathophysiology, target-organ involvement, treatment and challenges for future research. Journal of Bone and Mineral Research, 2011; 26(10): 2317-2337.
19. Velasco PJ, Manshadi M, Breen K, Lippman S. Psychiatric aspects of parathyroid disease. Psychosomatics, 1999; 40: 486-490.
20. Kowdley KV, Coull BM, Orwoll ES. Cognitive Impairment and Intracranial Calcification in Chronic Hypoparathyroidism. The American Journal of the Medical Sciences, 1999; 317(5): 273.
21. Centre for Reviews and Dissemination. Systematic reviews: CRD’s guidance for undertaking reviews in health care. University of York, 2009.
22. Downs SH, Black N. The feasibility of creating a checklist for the assessment of the methodological quality of both randomized and non-randomized studies of health care interventions. J Epidemiol Community Health, 1998; 52: 377-38.
